# Hand Book of Local Therapeutics

**Published:** 1893-10

**Authors:** 


A HANDBOOK OF LOCAL THERAPEUTICS.—By Allen, Harte, Harlan and Van Harlingen. Edited by Harrison Allen, M. D. Octavo, 500 pages. Price, $4.00. P. Blakiston, Son & Co., Philadelphia.

The need for a book of this character has long been apparent, for there has been no text available in which the *local action of drugs* was not subordinated to their general actions, while the average text-book omits altogether mention of many agents that in the hands of a specialist become valuable aids to cure.

Diseases which require chiefly local treatment are those of the respiratory passages, eye, ear, and skin, together with certain general surgical affections, including the diseases of women; it is, therefore, to the great advantage of the book that each remedy has been thoroughly set forth by different authors who have had large practical experience in these various branches.

Each remedy has been taken up in alphabetical order, and after a description of its pharmaceutical properties, is considered in reference to its physiological effect and value in local treatment.

The demands for thorough revision of local medicaments made by the advance of theories of asepsis, have been fully considered, and a succinct account has been presented of the source and properties of the very numerous new agents which affect tissues locally.

Some drugs have been excluded which have been highly praised; on the other hand great care has been taken not to indorse imperfectly attested novelties.

This hand-book embodies the results obtained by experienced teachers, and will prove a very valuable work to the general practitioner. Two carefully made indexes make it a book of ready reference.

